# Authors’ Reply to: Bibliometric Studies and the Discipline of Social Media Mental Health Research. Comment on “Machine Learning for Mental Health in Social Media: Bibliometric Study”

**DOI:** 10.2196/29549

**Published:** 2021-06-17

**Authors:** Jina Kim, Daeun Lee, Eunil Park

**Affiliations:** 1 Department of Applied Artificial Intelligence Sungkyunkwan University Seoul Republic of Korea

**Keywords:** bibliometric analysis, machine learning, mental health, social media, bibliometrics

We thank all comments and critical insights on our study, “Machine Learning for Mental Health in Social Media: Bibliometric Study” [1]. In this letter, we responded to the discussion points raised by Resnik and colleagues [2].

First, Resnik et al [2] pointed out the limited scope of the search query selected in our bibliometric study, which concealed the approaches taken in the clinical research field. We agree that the search query may seem to be lacking in covering clinical research; however, we did not intend to distort the recent trends in machine learning for mental health in social media. Moreover, considering that publication venues including the *Journal of Medical Internet Research, BMJ Open*, *International Journal of Environmental Research and Public Health, Frontiers in Psychiatry*, and *Frontiers in Psychology* were listed as productive publication sources in the analysis results, we believe that the publications employed in our analysis cover the invaluable research methodologies in the medical research area. The completed list of retrieved publications can be found in the appendix [1].

Moreover, Resnik et al [2] also stated that the machine learning–related terms in the search query lean toward specific technological methodologies, which may not include solutions including screening, risk assessment, and monitoring. We could not agree more with their point of view on the importance of practical works applied to “real” clinical settings. However, since such approaches directly related to diagnosis or treatment required careful consideration, we had difficulties fully diving into it. Thus, we want to stress that the main focus of our research was to help clinical researchers and field affiliates understand the technical needs of machine learning, which we believe is necessary for the initiation phase of this research field. Besides, we conducted a thorough trend review analysis of highly cited papers to provide a more in-depth understanding of related research fields [3].

We appreciate the suggestions on keywords for the search query to include “clinical psychology,” “psychiatry,” and “computational linguistics.” This led us to consider interviewing domain experts to set more domain-specific keywords in future research, following the procedure of the previous bibliometric study [4].

Third, Resnik and colleagues [2] raised concerns about only using indexing services and excluding search engines such as Google Scholar. Considering the rapidly changing trends in this research area, we employed both Web of Science and Scopus for the analysis to cover not only journal publications but conference proceedings. Nevertheless, we acknowledge Resnik and colleagues’ concern and also mentioned the need to employ other medical-oriented databases, which may provide more significant academic and practical information in the *Discussion* section. We would also like to take this opportunity to recap the importance of nonpublished work by sharing a preprint paper in this research field [5], which helped us to take a step toward future research on applying machine learning and social media in mental health counseling.

We hope our analysis may trigger all stakeholders to further consider how to employ machine learning approaches toward mental health in social media and be a support to bridge the gap between technologists and clinicians.
